# Evaluating requests for physician‐assisted suicide. A survey among German oncologists

**DOI:** 10.1002/cam4.4981

**Published:** 2022-06-30

**Authors:** Jan Schildmann, Marc Cinci, Leonie Kupsch, Michael Oldenburg, Bernhard Wörmann, Stephan Nadolny, Eva Winkler

**Affiliations:** ^1^ Institute for History and Ethics of Medicine, Interdisciplinary Centre for Health Sciences Medical Faculty of Martin Luther University Halle‐Wittenberg Halle (Saale) Germany; ^2^ Department of Medical Oncology, National Centre for Tumour Diseases, Section for Translational Medical Ethics University Hospital Heidelberg Germany; ^3^ German Society of Haematology and Medical Oncology (DGHO) Berlin Germany; ^4^ Department of Internal Medicine, Haematology/Oncology and Tumour Immunology Charité University Medicine Berlin Germany

**Keywords:** cross‐sectional studies, ethics, haematology, medical oncology, physician‐assisted suicide, survey

## Abstract

**Background:**

Cancer patients form a notable proportion of requestors for physician‐assisted suicide (PAS). This manuscript provides data on German oncologists' views concerning due criteria for the assessment of requests for PAS and quality assurance.

**Methods:**

The German Society of Haematology and Medical Oncology (DGHO) has conducted a survey among its members to elicit data about practices and views on regulating PAS in March 2021. Descriptive analysis and bivariate logistic regression of quantitative data on socio‐demographic and other determinants possibly associated with respondents' views on PAS as well as content analysis of qualitative data were performed.

**Results:**

About 57.1% (*n* = 425) of respondents (*n* = 745) indicated that they had been asked for information about PAS by patients. Information about palliative (92.7%; *n* = 651) and psychological care options (85.6%; *n* = 598) was deemed most important in cases of requests for PAS. More than half of the respondents (57.6%; *n* = 429) were in favour of a formal expert assessment of decisional capacity and about 33.4% (*n* = 249) favoured a time span of 14 days between the counselling and prescription of a lethal drug. There was no association between participants who received more requests and a preference for disclosing publicly their willingness to assist with suicide. A majority of respondents requested measures of quality assurance (71.3%; *n* = 531).

**Conclusion:**

According to respondents' views, the regulation of PAS will require diligent procedures regarding the assessment of decisional capacity and counselling. The findings suggest that the development of adequate and feasible criteria to assess the quality of practices is an important task.

## Graphical abstract

More than half of German oncologists have been asked for information about assisted suicide by their patients. In the wake of new laws in Germany and other countries this survey presents data on oncologists’ views regarding adequate regulation.

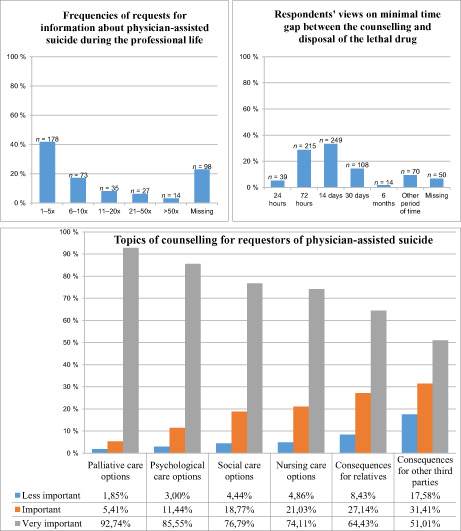

## 1 BACKGROUND

Assisted suicide is a rare and, at the same time, controversial end‐of‐life practice. (Physician‐)assisted suicide (PAS) has become legal in Germany and several other countries more recently. However, the legal frameworks of those countries which allow PAS differ regarding a range of aspects such as requirements for eligibility for PAS, assessment of decision capacity and further due criteria.^1^, ^2^, ^3^, ^4^

Regarding oncological clinical practice, PAS and its regulation are relevant topics for several reasons. Firstly, a proportion of patients with cancer consider ending their lives during the course of their disease.^5^, ^6^, ^7^ Secondly, most requests for PAS stem from patients with cancer.^8^, ^9^, ^10^ Thirdly, oncologists routinely prescribe drugs which could potentially be used for suicide as part of a patient's treatment for pain and other symptoms.

Subsequent to a controversial debate, the German parliament decided to prohibit the support of assisted suicide by § 217 of the penal code in 2015. The normative framework relevant to assisted suicide changed again recently as the German Federal Constitutional Court deemed criminalisation of assisted suicide services unconstitutional in February 2020. According to this judgement, ‘The general right of personality (Art. 2(1) in conjunction with Art. 1(1) of the Basic Law, Grundgesetz – GG) encompasses a right to a self‐determined death. This right includes the freedom to end one's own life and, as the case may be, resort to assistance provided voluntarily by third parties for this purpose’.^11^ As a consequence, the German Medical Association withdrew the prohibition of PAS as part of the professional code in May 2021.

Current propositions to regulate PAS by law^12^, ^13^, ^14^, ^15^ differ regarding the criteria for evaluating requests for assisted suicide, the role of physicians and recommended measures for quality assurance. Given the relevance of such criteria for evaluating requests and the need for a framework which supports the high quality of care in cases of requests for assisted suicide, the German Society of Haematology and Medical Oncology (DGHO) has conducted a survey among its members to elicit data about practices and views on regulating PAS. In this paper, we present findings on the views of DGHO members regarding due care criteria for evaluating requests for PAS and measures of quality assurance. The aims of this study are

1. to describe respondents' views concerning the evaluation of requests for PAS
2. to elicit and analyse data about measures perceived to be relevant for quality assurance regarding the practice of PAS
3. to analyse socio‐demographic and other determinants which may be associated with respondents' views

## 2 METHODS

Development of the questionnaire has been reported elsewhere^16^ and is based on earlier studies.^17^, ^18^, ^19^, ^20^, ^21^ The format of the questions included multiple‐choice answers along with options for free‐text comments. Precursory versions of the survey were pre‐tested by researchers and research students.

The DGHO distributed the anonymous convenience‐sampled, cross‐sectional online survey together with a short invitation e‐mail to all members of the DGHO on 11 March 2021, with two ensuing reminders, 1 and 2 weeks later, respectively. The survey was closed on 31 March 2021. The study received exempt voting by the research ethics committee of the Medical Faculty of the Martin Luther University Halle‐Wittenberg.

Results of the descriptive analysis are provided as total numbers and percentages. Answers provided in free‐text fields were coded independently by two researchers with a background in medicine (LK, MC) according to thematic qualitative analysis grounded in hermeneutics.^22^ One‐third of the data was inductively coded followed by a session to develop a consented, data‐derived coding scheme. The remaining data were then analysed with the coding scheme with the possibility of adding subcodes if necessary. Since there was a lack of depth to the data, we opted for reporting in absolute frequencies. Categorisation as a result of the qualitative analysis of all free‐text comments is provided in a supplement of this manuscript.

Prior to statistical analysis and based on factors which have been described as relevant regarding end‐of‐life decision‐making,^23^, ^24^, ^25^, ^26^ we formulated the following hypotheses to further understand the distribution of answers among specific groups:

- Respondents who reported more than 10 requests for information were significantly more in favour of publicly available information on physicians willing to assist with suicide
- Respondents who reported more than 10 requests for information more frequently demanded an expert assessment of decisional capacity
- Female respondents rated information about consequences on relatives or other third parties as more important than others
- Female respondents considered more topics as important regarding counselling than others

Binary logistic regression was used to explore bivariate relationships between the dependent and independent variables for hypotheses a–c. For hypothesis d an independent *t*‐test was performed. Odds ratios (ORs), mean differences (MD) and their 95% confidence intervals (CIs) were computed. Statistical analysis was performed with IBM SPSS Statistics version 24.0 for Windows.

## 3 RESULTS

As described elsewhere,^16^ 745 of 3588 DGHO members responded to the survey, resulting in a response rate of 20.8%. According to reported respondent data, one oncology nurse and 28 people not (currently) working clinically in oncology participated. Regarding gender, age and place of work, the sample of respondents represents the socio‐demographic structure of all members of the society except for a higher proportion of respondents who had an additional qualification in palliative care.^16^ Table 1 summarises the detailed socio‐demographic data of respondents and of all DGHO members (adapted version, for the original publication of data, see Ref. [16]).

**TABLE 1:** Socio‐demographic characteristics (respondents: *n* = 745, deviations from 100% result from missings in the respective variable)

	Respondents: *n* (%)	DGHO members overall: *n* (%)
Gender		
Female	272 (36.5)	1309 (35.7)
Male	420 (56.4)	2.360 (54.3)
Diverse	4 (0.5)	Not available
Age		
<30	18 (2.4)	59 (1.6)
30–40	92 (12.4)	501 (13.7)
41–50	166 (22.3)	823 (22.4)
51–60	245 (32.9)	958 (26.1)
>60	172 (23.1)	685 (18.7)
Median	51–60 years	51–60 years
Professional experience		
<10 years	89 (11.9)	Not available
>10 years	599 (80.3)	Not available
Mean	21.5 years	Not available
Median	21.0 years	Not available
Workplace		
Outpatient	277 (37.2)	881 (24.0)
Inpatient	458 (61.5)	1.964 (53.5)
a) University hospital	207 (27.8)	Not available
b) Other hospital	51 (33.7)	Not available
Other	61 (8.2)	824 (22.5)
Specialisation in palliative care		
Yes	344 (46.2)	909 (24.8)
No	356 (47.8)	2760 (75.2)

### 3.1 Frequency of requests for information

**FIGURE 1:**
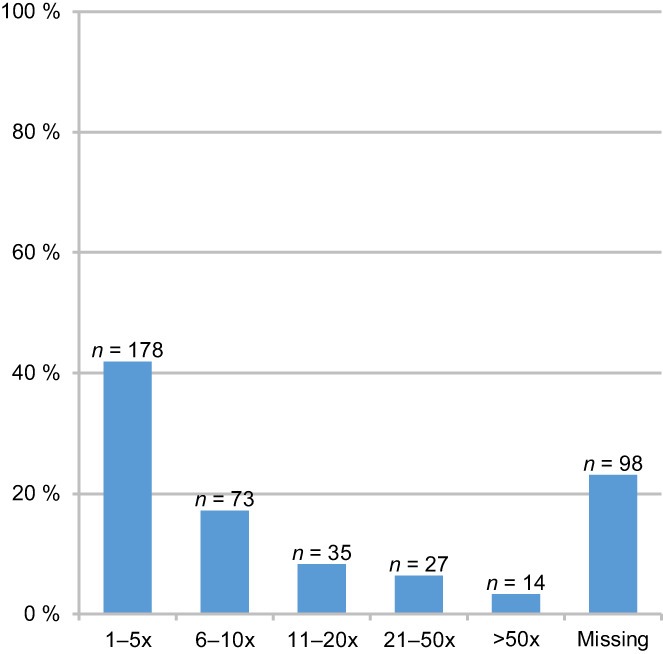
Frequencies of requests for information about physician‐assisted suicide during the professional lifetime (recoded and grouped figures given as free text, answered by *n* = 425).

More than half of the respondents (57.1%; *n* = 425) indicated that they had been asked for information about PAS. According to respondents of this subgroup, these requests happened between once and over 50 times within their professional life so far. Figure 1 indicates the distribution of frequencies of requests for information during the professional life.

### 3.2 Content of counselling and process of evaluating requests for physician‐assisted suicide

**FIGURE 2:**
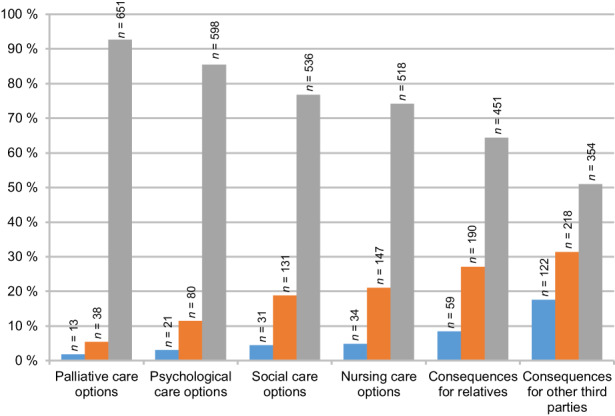
Topics of counselling for requestors of physician‐assisted suicide (multiple answers possible; answered by *n* = 705 respondents, missing: *n* = 40; blue = less important, orange = important, grey = very important).

Survey participants were shown a list of possible topics to be considered as part of the counselling of patients requesting PAS. Information about palliative (92.7%; *n* = 651) and psychological care options (85.6%; *n* = 598) and social work (76.8%; *n* = 536) were deemed most important. In addition to pre‐selected topics, respondents could indicate further topics in a free‐text field. Based on the content analysis of these answers, eight respondents referred to ‘spiritual issues’ as a topic for counselling, five mentioned ‘possible complications and risks associated with assisted suicide’ and three respondents suggested ‘counselling regarding legal issues’. Figure 2 summarises the findings on possible counselling topics and their respective importance as viewed by respondents.

More than half of the participants (57.6%; *n* = 429) were in favour of a formal expert assessment of decisional capacity for all requestors of PAS, whereas 32.8% (*n* = 244) thought such a requirement was necessary only in some cases. Of the latter group, 89.8% (*n* = 219) were in favour of a formal assessment by an expert if patients had a mental health diagnosis and 84.8% (*n* = 207) in cases in which requesting patients had a good prognosis. Ten respondents mentioned psychosocial challenges, nine brought up ‘age under 18 years’ and seven respondents referred to doubts regarding decisional capacity as additional reasons for formal expert assessment. Furthermore, five respondents mentioned the ‘need for clear regulatory criteria for the assessment of decisional capacity’ and four participants referred to the ‘need for a simple procedure’ regarding the assessment of decisional capacity in a field for free‐text comments related to the question.

**FIGURE 3:**
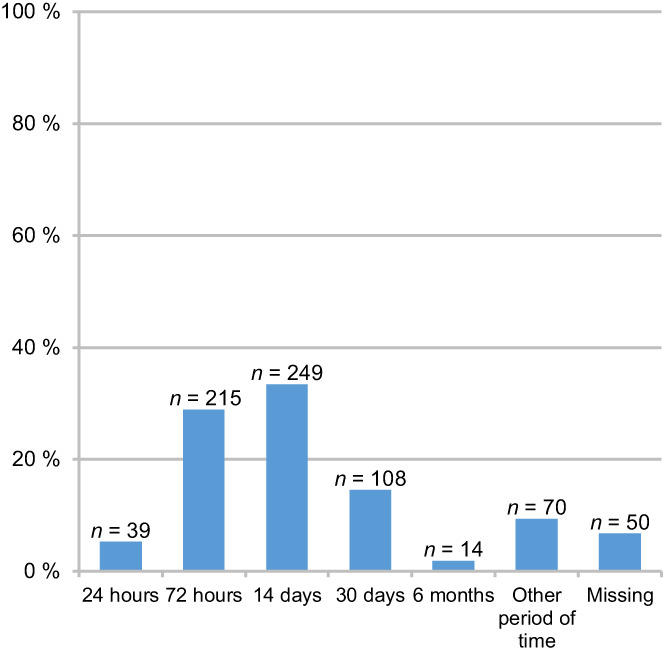
Respondents' views on a minimum time gap between the counselling and disposal of the lethal drug (single answer option; answered by *n* = 695 respondents).

Regarding patients with cancer, 33.4% (*n* = 249) of respondents favoured a time span of 14 days minimum between the counselling and prescription of the lethal drug, whereas 28.9% (*n* = 215) considered a time span of 72 h minimum to be sufficient. In addition to pre‐selected time spans, respondents were also able to answer the question by means of free‐text answers. Twenty‐three respondents made statements ‘objecting to general time spans/pleading for individual solutions’. Figure 3 provides an overview of respondents' views regarding pre‐selected minimum time spans between the counselling and the disposal of the lethal drug.

### 3.3 Implementation and quality assurance

Asked about possible ways to implement assisted suicide in practice, 25.8% (*n* = 192) favoured dispensing drugs with the possibility of committing suicide at the time and place of the person's choosing, whereas 23.9% (*n* = 178) preferred a model according to which the time and place of suicide were arranged beforehand. About a quarter of the respondents (26.3%; *n* = 196) rejected both options and 18.1% (*n* = 135) were undecided. Sixteen respondents mentioned the ‘importance of an individual choice of place of death’ in free‐text comments, whereas 11 study participants referred to ‘measures needed to monitor the process’ until the death of the person. In addition, nine respondents mentioned ‘regulation of circumstances of death’, for example, regarding the impact on others possibly encountering a person after suicide.

A majority of respondents supported measures of quality assurance (71.3%; *n* = 531) as part of the legal regulation of assisted suicide. Within this group of respondents, about equal proportions supported educational measures for physicians (75.5%; *n* = 474), an obligation to report the disposal of drugs (70.7%; *n* = 444), an obligation to report counselling a requestor (69.3%; *n* = 435) and accompanying research (69.3%; *n* = 435). Analysis of additional free‐text comments showed that 10 respondents viewed ‘documenting the process’ as an important quality measure, whereas the answers of six respondents each focused on ‘interdisciplinary decision‐making’ and the ‘involvement of an ethicist’ as further measures of quality assurance.

More than half of the respondents were in favour of physicians willing to assist with suicide and also being able to publicly disclose their services (52.0%; *n* = 387), whereas 40.5% (*n* = 302) were against publicly available information. Of those who supported public information about offering assistance for suicide, a majority voted for the regulation of such information (82.9%; *n* = 325) and providing it only in combination with information on suicide prevention (56.4%; *n* = 221). A minority of respondents (8.7%; *n* = 34) preferred giving out information about assisted suicide without any limitations. In addition to pre‐selected answer options, 11 respondents commented in the form of free text on the need for ‘information provided by health professionals only’. Comments of eight respondents referred to ‘official sources of information’ as adequate tools for information about the topic. Six study participants distinguished ‘neutral information’ as preferable to ‘advertising information’ in their comments.

### 3.4 Determinants associated with requests for information and views on regulation

The following results were obtained regarding the hypotheses established. There was no association between participants who received more than 10 requests for information about assisted suicide and a preference for disclosing publicly which physicians were willing to assist with suicide (OR: 1.19; CI: 0.74–1.93) or the demand for expert assessment of decisional capacity (OR: 1.53; CI: 0.33–13.43). Women did not consider information about consequences for relatives (OR: 0.91; CI: 0.52–1.57) or third parties (OR: 1.21; CI: 0.81–1.82) more important than others. Furthermore, female respondents did not consider more topics as important regarding counselling than other respondents (MD: 0.13; CI: 0.03–0.29).

## 4 DISCUSSION

This paper provides data on oncologists' views on due diligence criteria relevant to the evaluation of requests for PAS and aspects of quality assurance in the wake of establishing a regulatory framework in Germany. The main findings are, firstly, the high number (57%) of respondents with requests for information about PAS and the majority vote (58%) for an obligatory formal expert assessment of the decisional capacity. Secondly, 14 days as the minimum time span between evaluating a request of a cancer patient and the disposal of lethal drugs was considered to be sufficient by the largest group of respondents (33%). Thirdly, half of the respondents supported publicly available information on physicians who offer to assist in suicide. Fourthly, a majority (71%) supported measures of quality assurance.

### 4.1 Evaluation of requests and counselling

A majority of respondents (57%) had at least one request regarding information about PAS, though there are notable differences regarding the frequencies of these requests indicated. In our earlier study conducted in 2015 with members of DGHO, 43% of the respondents indicated that they had been asked by their patients whether they would be willing to assist in suicide.^18^ Comparison of these data with other studies is difficult due to the different wording of related questions. In a previous study among US oncologists, half of the respondents reported requests for assisted suicide.^27^

Respondents named a range of topics, such as palliative care, and psychological and social support, which they deemed as important in the context of counselling. This begs the question according to which procedure such comprehensive information will be communicated as part of the counselling process and who will be responsible for providing it. Furthermore, one should bear in mind that there may be differences regarding the information relevant for different groups of requestors of PAS (e.g. cancer patients with a life‐limiting disease versus older people who do not want to live longer).

Most respondents favoured a formal assessment of decisional capacity by an expert for all requestors. In this respect, some authors have demanded the involvement of psychiatrists to formally assess decisional capacity as part of the evaluation of requests for PAS.^28^ An important task for researchers will be the development and validation of instruments for assessing decisional capacity which can be used for a potentially increasing number of patients and healthy citizens who may request PAS. A number of instruments for capacity assessment have been developed,^29^ however, it is not clear whether those can also be used for the specific situation of requesting PAS. In this respect, it will be important not ‘to err on the wrong side’ in terms of failing to notice a lack of decisional capacity in patients requesting PAS; it is also necessary to avoid setting the bar so high that it will, in fact, be impossible to meet the criteria for decisional capacity.^30^ Furthermore, it should be noted that, depending on the regulatory framework, capacity at the time of the request may not imply that a person still has decisional capacity when ingesting the lethal drug.^3^

A majority of respondents deemed a time span of 14 days between counselling requesting cancer patients and providing the means for suicide as sufficient. On the one hand and in light of data showing that wishes for hastening death can fluctuate in patients with advanced cancer, this seems rather short (for a review of data, see Ref. [31]). In addition, some requests might be associated with clinical depression^5^, ^7^ and a longer time span combined with a trial of antidepressants or palliative care treatment may contribute to patients reconsidering a request for PAS. On the other hand, the responses of survey participants might be framed by personal experiences with patients having a poor prognosis. This interpretation is supported by our qualitative analysis of comments, which shows that a proportion of respondents object to predetermined time spans and emphasise the need for making individual decisions regarding the time span between a request and the provision of the lethal drug.

### 4.2 Implementation and quality assessment

As indicated, there are currently different models for implementing PAS. In Oregon, patients may choose the time and place of death after having been prescribed the lethal drug, whereas in Switzerland, patients and representatives of respective organisations usually agree on both time and place of death.^3^, ^4^ These and further differences regarding the practicalities have far‐reaching consequences regarding planning ahead, for example concerning the involvement of relatives, but also safety issues regarding the storage of lethal drugs. The heterogeneity of responses regarding support for one of the models mentioned above or none indicates that while there is already a divide on principle issues of PAS, it seems even more challenging to develop a practical framework which is likely to be accepted by a considerable proportion of oncologists.

One option to steer and adapt the mode of implementation of PAS subsequent to its legislation may be a stringent programme of quality assurance which is endorsed by a large majority of participants. Documentation of the process and scientific evaluation including follow‐up may provide a sound basis for potentially necessary adjustments regarding the framework. However, it should also be noted that collecting such information needs to be feasible to encourage and facilitate the reporting of the actual practice. In addition, it will be necessary to select criteria which are both valid regarding the quality of the practice and empirically robust. From an ethical perspective, gathering and reporting quality indicators is important to increase the transparency and further trust of society in a healthcare system which needs to deal with the rare but highly controversial practice of PAS.

### 4.3 Limitations

A limitation of this study is the response rate of 20.8% and the reliance on convenience sampling. Accordingly, the survey may present findings of a group of oncologists particularly interested in the topic. A possibility of unit non‐response bias could also lie with professionals who are particularly opposed to PAS. Furthermore and given that the society mailing list also included a small number of members who were not oncologists, not all reported data stem from oncologists. However, the number of respondents identifying themselves as oncologists with clinical experience in each relevant question allows for a robust data analysis and the socio‐demographic characteristics are comparable with the overall sampling frame, which was the DGHO member list.^16^ Another factor possibly limiting the interpretation of findings is the wording of the questions. We considered this factor as part of the pre‐test by involving practitioners, researchers and students with different moral stances towards the topic to avoid judgemental language as much as possible. Another limitation is that information given in free‐text comments had to be recoded by researchers to be presented in this manuscript. While this process was conducted by two researchers independently, codes might differ if analysed by other researchers. Finally, social expectations may have influenced the respondents' answers.

## 5 CONCLUSIONS

The quantitative and qualitative data of this non‐representative survey of German oncologists' views on assessing requests for PAS suggest that a framework to regulate the controversial practice needs to balance a multitude of aspects which may partially be difficult to reconcile. This is particularly true for requirements which, on the one hand, support the good practice of assessing decisional capacity and counselling and, on the other hand, are feasible and do not set the bar so high that it becomes impossible for patients to realise their decision of ending their lives. In addition, the findings suggest that the development of adequate, empirically robust and feasible criteria to assess the quality of practices related to (requests for) PAS is an important task for those countries in which PAS is an option.

## AUTHOR CONTRIBUTIONS

Jan Schildmann: Conceptualisation, methodology, investigation, writing ‐ original draft, review & editing, supervision. Marc Cinci: Formal analysis, writing ‐ review & editing. Leonie Kupsch: Formal analysis, writing ‐ review & editing. Michael Oldenburg: Methodology, writing ‐ review & editing. Bernhard Wörmann: Methodology, writing ‐ review & editing. Stephan Nadolny: Formal analysis, resources, writing ‐ review & editing. Eva Winkler: Conceptualisation, methodology, writing ‐ original draft, review & editing.

## FUNDING INFORMATION

None declared.

## CONFLICT OF INTEREST

The authors have declared no conflicts of interest.

## ETHICS STATEMENT

The study received exempt voting by the research ethics committee of the Medical Faculty of the Martin Luther University Halle‐Wittenberg (Reg. No. 2021–054).

## Supporting information


Appendix S1

## Data Availability

The data that support the findings of this study are available from the corresponding author upon reasonable request.
