# Supplementary material for: Evaluating requests for physician‐assisted suicide. A survey among German oncologists
Source: Cancer Med. 2022 Jun 30;12(2):1813–20. doi: 10.1002/cam4.4981 (PMC9883542; doi:10.1002/cam4.4981)
Supplement: Supplementary file 1 — Appendix S1 [file CAM4-12-1813-s001.pdf]

Supplement: Qualitative analysis – categorization of free text comments related to data presented (authors' translation)

S1: *Question:* How important do you consider counselling on the following topics to be in the event of legal regulation of (physician-) assisted suicide?

Categories	Absolute number of participants (*n*) with quote allocated to category
Spiritual issues	8
Possible complications and risks associated with assisted suicide	5
Counselling regarding legal issues	3
Impact on physicians	2
Comments that do not fit into any category	8

S2: *Question:* In which cases should an expert opinion (on decisional capacity) be made.

Categories	Absolute number of participants (*n*) with quote allocated to category
Psychosocial challenges	10
Age under 18 years	9
Doubts regarding decisional capacity	7
Differences of opinion on whether a patient has decisional capacity	3
Comments that do not fit into any category	5

S3: *Question:* In case of legal regulation on assessment of decisional capacity, an expert opinion should be requested for (which patients).

Categories	Absolute number of participants (*n*) with quote allocated to category
Need for clear regulatory criteria for the assessment of decisional capacity	5
Statements against physician-assisted suicide	5
Need for a simple procedure	4
Ambiguities about the research question	3
Comments that do not fit into any category	13

S4: *Question:* In case of legal regulation on counselling in the context of (physician-) assisted suicide, the minimum time period after counselling of patients with cancer should be at least….

Categories	Absolute number of participants (*n*) with quote allocated to category
Objecting to general time spans/plea for individual solutions	23
1 week	21
> 1 month but less than 6 months	11
Unlimited time span	3
Rejecting implementation of regulations	2
Comments that do not fit into any category	8

S5: *Question:* Assisted suicide has been implemented differently in those countries where it is legally possible. What approach, if any, do you consider appropriate?

Categories	Absolute number of participants (*n*) with quote allocated to category
Importance of individual choice of place of death	16
Measures needed to monitor the process	11
Regulation of circumstances of death	9
Hospital or palliative care setting	4
Statements against physician-assisted suicide	3
Comments that do not fit into any category	15

S6: *Question:* Which quality assurance measures should be implemented?

Categories	Absolute number of participants (*n*) with quote allocated to category
Documenting the process	10
Interdisciplinary decision-making	6
Involvement of an ethicist	6
Professional training/qualification necessary for all personnel involved	5
Psychological support for all personnel involved	4
Feedback on outcome and possible complications	4
Supervision of personnel involved in decision-making	3
Exhausting all alternative options	2
Comments that do not fit into any category	11

S7: *Question:* Doctors (or institutions) who offer assisted suicide should be allowed to provide information about their services.

Categories	Absolute number of participants (*n*) with quote allocated to category
Information provided by health professionals only	11
Official sources of information	8
Statements against physician-assisted suicide	7
Neutral information, no advertising for physician-assisted suicide	6
Regulations should be similar to laws pertaining abortion	3
Comments that do not fit into any category	20
